# Circadian rhythm in systemic autoimmune conditions: Potential of chrono-immunology in clinical practice: A narrative review

**DOI:** 10.1097/MD.0000000000034614

**Published:** 2023-08-11

**Authors:** Wireko Andrew Awuah, Helen Huang, Jacob Kalmanovich, Aashna Mehta, Tatiana Mikhailova, Jyi Cheng Ng, Toufik Abdul-Rahman, Favour Tope Adebusoye, Joecelyn Kirani Tan, Karl Kamanousa, Tomas Ferreira, Sakshi Roy, Mrinmoy Kundu, Rohan Yarlagadda, Nobendu Mukerjee, Athanasios Alexiou, Marios Papadakis

**Affiliations:** a Faculty of Medicine, Sumy State University, Sumy, Ukraine; b Royal College of Surgeons in Ireland, University of Medicine and Health Sciences, Dublin, Ireland; c Drexel University College of Medicine, Philadelphia, PA; d University of Debrecen-Faculty of Medicine, Debrecen, Hungary; e SUNY Upstate Medical University, Syracuse, NY; f Faculty of Medicine and Health Sciences, University of Putra Malaysia, Serdang, Malaysia; g Faculty of Medicine, University of St Andrews, Scotland, UK; h School of Clinical Medicine, University of Cambridge, Cambridge, UK; i School of Medicine, Queen’s University Belfast, Belfast, UK; j Institute of Medical Sciences and SUM Hospital, Bhubaneswar, India; k Rowan University School of Osteopathic Medicine, Stratford, NJ; l Department of Microbiology, West Bengal State University, Barasat, India; m Department of Health Sciences, Novel Global Community Educational Foundation, Hebersham, NSW; n Department of Science and Engineering, Novel Global Community Educational Foundation, Hebersham, NSW; o Department of Surgery II, University Hospital Witten-Herdecke, Heusnerstrasse 40, University of Witten-Herdecke, Wuppertal, Germany.

**Keywords:** chrono-immunology, circadian rhythm, systemic autoimmune diseases

## Abstract

The circadian rhythm (CR) is a fundamental biological process regulated by the Earth’s rotation and solar cycles. It plays a critical role in various bodily functions, and its dysregulation can have systemic effects. These effects impact metabolism, redox homeostasis, cell cycle regulation, gut microbiota, cognition, and immune response. Immune mediators, cycle proteins, and hormones exhibit circadian oscillations, supporting optimal immune function and defence against pathogens. Sleep deprivation and disruptions challenge the regulatory mechanisms, making immune responses vulnerable. Altered CR pathways have been implicated in diseases such as diabetes, neurological conditions, and systemic autoimmune diseases (SADs). SADs involve abnormal immune responses to self-antigens, with genetic and environmental factors disrupting self-tolerance and contributing to conditions like Systemic Lupus Erythematosus, Rheumatoid Arthritis, and Inflammatory Myositis. Dysregulated CR may lead to increased production of pro-inflammatory cytokines, contributing to the systemic responses observed in SADs. Sleep disturbances significantly impact the quality of life of patients with SADs; however, they are often overlooked. The relationship between sleep and autoimmune conditions, whether causal or consequential to CR dysregulation, remains unclear. Chrono-immunology investigates the role of CR in immunity, offering potential for targeted therapies in autoimmune conditions. This paper provides an overview of the connections between sleep and autoimmune conditions, highlighting the importance of recognizing sleep disturbances in SADs and the need for further research into the complex relationship between the CR and autoimmune diseases.

## 1. Introduction

The circadian rhythm (CR), also known as the sleep and wake cycle, plays a critical role in maintaining the body’s homeostatic mechanisms. The suprachiasmatic nucleus (SCN), located in the hypothalamus, regulates the brain’s internal clock, which operates on a 24-hour cycle. The CR is responsible for controlling wakefulness and drowsiness by responding to light variations in the environment.^[1]^ This regulation is facilitated by feedback loops involving “clock genes” such as brain and muscle ARNT-like protein-1-2 (BMAL1-2), Circadian Locomotor Output Cycles Kaput (CLOCK), period circadian regulator 1-3 (PER1-3), and cryptochrome circadian regulator 1-2 (CRY1-2).^[2]^ When exposed to light, the SCN receives signals from retinal ganglion cells, leading to reduced melatonin production by the pineal gland through the suppression of the paraventricular nucleus and sympathetic nervous system mediated by gamma-aminobutyric acid.

At night, in the absence of light, the SCN resumes melatonin production, promoting sleep. This cyclical process affects multiple systems, including the immune system.^[1]^ The circadian oscillations of cytokines, hormones, and other effector molecules regulate the immune system’s ability to defend against various illnesses. Disruptions in the CR have been associated with immune disorders, particularly systemic autoimmune diseases (SAD).^[3]^

SADs encompass a wide range of conditions characterized by loss of self-tolerance and heightened immune system activity against self-antigens, resulting in widespread damage to various tissues and organs.^[4]^ Currently, SADs affect approximately 400,000 individuals worldwide.^[5]^ This review will focus on major systemic inflammatory disorders, namely Rheumatoid Arthritis (RA), Systemic Lupus Erythematosus (SLE), and Inflammatory Myopathies (IM). SADs tend to have a higher prevalence in women, with Sjögren syndrome, SLE, and antiphospholipid syndrome exhibiting the most significant gender imbalances.^[6]^ Each SAD primarily affects specific age groups.^[6,7]^ Kawasaki disease, Henoch-Schonlein Purpura, and conditions associated with primary immunodeficiencies are the most common SADs in children.^[6]^ Young adults are more prone to SLE, sarcoidosis, and Behçet’s disease, while Sjögren syndrome is more prevalent among middle-aged individuals.^[6]^ Amyloidosis, polymyalgia rheumatica, and giant cell arteritis primarily affect the elderly.^[7]^

The loss of self-tolerance associated with SADs is believed to have a multifactorial etiology involving genetic, epigenetic, and environmental factors.^[8–10]^ Environmental factors such as microbes, ultraviolet light exposure, diet, and toxins can increase the likelihood of disease occurrence in individuals with a genetic predisposition.^[9,10]^ Autoimmunity is thought to be induced by excessive inflammatory responses triggered by common infections. However, this process is not yet fully understood and involves various contributing factors.^[8,10]^ Symptom manifestation occurs when autoimmunity leads to local tissue inflammation. Tissue destruction may result from antibody-mediated damage, immune complex deposition, or prolonged inflammatory responses. Epigenetic changes can also influence the development of systemic inflammatory conditions, leading to abnormal immune gene expression and the overproduction of major inflammatory molecules such as interferons (IFNs), tumor necrosis factors (TNFs), and interleukins (ILs).^[8,10]^ The interplay between the CR and immune genes is evident in SADs, with the CR playing a role in the disease’s pathophysiology.^[5,11]^ Additionally, the disease itself can affect the CR, creating a reciprocal relationship.^[5,11]^ Despite experimental evidence suggesting that the CR regulatory system may contribute to the development of autoimmune conditions, sleep disturbances observed in patients with SADs are not routinely prioritized in therapeutic management.^[12]^ Furthermore, the diagnostic criteria for SADs typically include only classic signs of inflammation, such as fever, pain, and elevated inflammatory markers, despite the potential importance of CR changes in disease progression.^[12]^ This review aims to highlight the significance of the CR in managing SADs and describes the most promising novel therapeutic targets.

## 2. Methodology

This narrative review provides a comprehensive overview of the circadian rhythm in systemic autoimmune conditions. The review included full-text articles written in English, without strict date limits on the included study publications. To ensure a thorough literature search, multiple databases were employed, including PubMed, EMBASE, Google Scholar, the Cochrane Library, and Scopus. The search terms used in combination were “circadian rhythm,” “sleep,” “sleep deprivation,” and “chrono-immunology,” along with relevant terms such as “systemic autoimmune conditions,” “rheumatoid arthritis,” “systemic lupus erythematosus,” and “inflammatory myopathies.” Studies involving both pediatric and adult populations were included to provide a comprehensive analysis.

Additionally, additional sources were identified through a manual search of references cited in recent reviews focused on the role of circadian rhythm in systemic autoimmune conditions. Stringent exclusion criteria were applied, which excluded standalone abstracts, case reports, posters, and unpublished or non-peer-reviewed studies. These criteria were implemented to ensure the inclusion of high-quality and reliable evidence.

The review did not set a predetermined limit on the number of studies to be included, aiming to gather a comprehensive understanding of the subject matter. Various study designs were encompassed, including descriptive studies, cohort studies, and observational studies. Both pre-clinical and clinical investigations were considered, providing a broad perspective on the impact of circadian rhythm in systemic autoimmune conditions. A summary of the methodology employed is presented in Table 1.

**Table 1:** Summary of methodology for circadian rhythm in systemic autoimmune conditions.

Methodology	Description
Literature search	PubMed, EMBASE, Google Scholar, the Cochrane Library, and Scopus.
Inclusion criteria	Full-text articles published in English published till date. Various study designs, such as descriptive studies, cohort studies, and observational studies. Studied involving pediatric and adult populations.
Exclusion criteria	Stand-alone abstracts and unpublished studies. Non-English studies.
Search terms	Keywords include “circadian rhythm” “sleep,” “sleep deprivation,” and “chrono-immunology.” Combined with relevant terms such as “systemic autoimmune conditions,” “rheumatoid arthritis,” “systemic lupus erythematosus,” and “inflammatory myopathies.”
Additional search	A manual search was conducted to find references for recently published, disease-specific reviews.
Sample size requirement	No strict sample size requirement.

## 3. Results and discussion

### 3.1. The role of circadian rhythm in sleep and immunological responses

**Figure 1.**
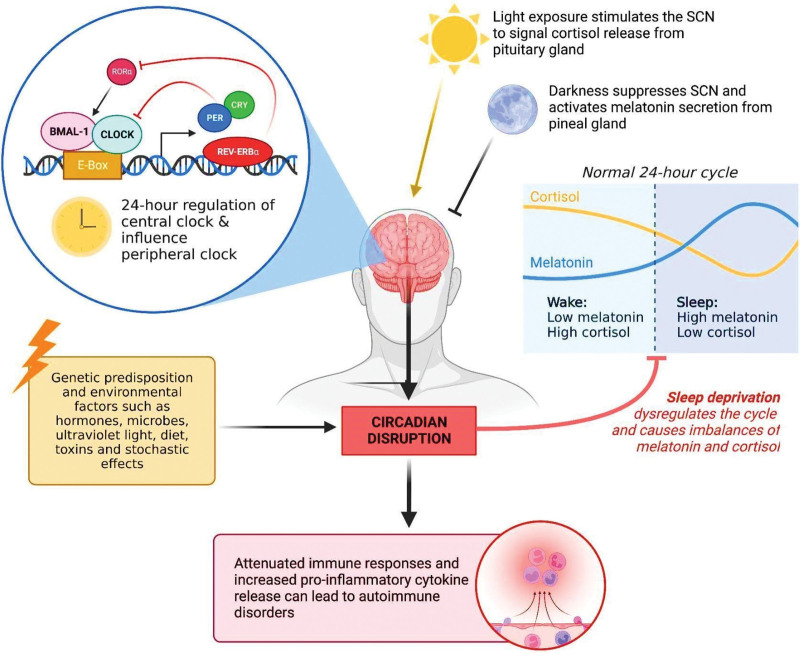
The mechanism of circadian regulation in the suprachiasmatic nucleus and its general effects on immune cell responses and sleep deprivation (Original figure created with biorender.com by HH). BMAL-1 = brain and muscle ARNT-like 1, CLOCK = circadian locomotor output cycles kaput, CRY = cryptochrome circadian regulator, PER = period circadian regulator, Rev-Erb = reverse orientation c-ErbA gene α, ROR = retinoic acid-related orphan receptor, SCN = suprachiasmatic nucleus.

The CR in mammals serves as an intrinsic system that regulates not only wakefulness and behavior but also intracellular homeostatic signaling. The CR contains central clocks/regulators in the suprachiasmatic nucleus (SCN), which governs several other regulatory pathways. It is important to note that this system is not limited to brain cells but influences the regulatory gene expression of all cell types.^[13]^ Experimental evidence has further demonstrated that even in the absence of sunlight, the circadian period in mammals is based on a 24-hour cycle, indicating the presence of an endogenous CR homeostasis maintained by conserved regulatory pathways.^[13]^ The CR is largely influenced by a 24-hour cycle of transcriptional feedback loops, where the transcription of genes is regulated by their protein products^[14]^ (Fig. 1). The CLOCK and BMAL1 proteins play a key role in this feedback regulation. These proteins form complexes with enhancer box (E-box) motifs within specific promoter regions, initiating the transcription of clock genes such as PER1-3 and CRY1-2.^[14]^ At a specific point, the resulting clock proteins can inhibit the CLOCK-BMAL1 dependent transcription of PER and CRY genes, establishing a negative feedback loop.^[14]^

Sleep, an important contributor to an individual’s health, is essential for the maintenance of immune responses. The sleep cycle is strictly regulated by CR genes. Orexin is an important signaling molecule, and is crucial for the sleep/wakefulness cycle. Orexin-producing neurons in the lateral hypothalamus are active during wakefulness.^[14]^ These neurons project to various sites in the midbrain and hindbrain, stimulating wake-promoting neurons that release neurotransmitters such as glutamate, histamine, dopamine, and acetylcholine.^[14]^ Yoshida et al determined that orexin levels in mouse models follow a 24-hour cycle, with levels increasing during the dark hours and decreasing throughout the day.^[15]^ The SCN has also been found to regulate this process.^[16]^ In a study by Zhang et al, mice with ablated SCNs and control mice were exposed to 3 different light conditions: 12-hour light and 12-hour dark, constant light, and constant dark. Orexin levels were measured every 4 hours. The control mice exhibited significant diurnal fluctuations in orexin concentrations under all conditions, whereas the SCN-ablated mice showed no rhythmic fluctuation.^[16]^

The CR has been found to exert influence on the immune system (Fig. 1) and exhibits a bidirectional relationship. Leukocytes demonstrate a cyclic pattern, which is achieved through the regulatory feedback of clock genes within the cells, accounting for the observed circadian variation. CR genes regulate pathogen recognition and cytokine secretion.^[17,18]^ Guerro-Vargas et al demonstrated the involvement of the SCN in the regulation of inflammatory responses. Mice with an absent SCN exhibited an extreme and lethal proinflammatory response to stimuli.^[19]^ In their study, lipopolysaccharide (LPS) from gram-negative bacteria was used to induce a proinflammatory response. A cycle of susceptibility to inflammatory stimuli was observed by administering LPS at different times of the day. During active periods, increases in temperature coincided with the release of proinflammatory cytokines such as tumor necrosis factor alpha (TNF-α) and IL-6.^[19]^

### 3.2. The effects of sleep deprivation on the progression of autoimmune diseases

Sleep disturbance is frequently reported among patients with SADs.^[20]^ For example, studies found that sleep disorders were prevalent in 55% to 85% of SLE patients and were associated with disease activity, age, pain, and psychological factors such as depression.^[21,22]^ There appears to be a bidirectional relationship between circadian disturbances and autoimmune diseases. A nationwide study revealed a 70% higher risk of developing autoimmune disorders among patients with chronic insomnia.^[23]^ Another large population-based cohort study involving 144,396 patients showed that sleep deprivation increases the risk of SLE development.^[24]^ Other studies have observed a higher incidence of autoimmune diseases in non-apnea sleep disorders,^[25]^ and relatives of SLE patients with a sleep duration of less than 7 hours per night have a higher likelihood of transitioning to SLE in the future.^[12]^ However, a recent study did not establish a causal relationship between genetically predicted traits (e.g., chronotype, sleep duration, insomnia, and daytime sleepiness) and SLE.^[26]^ Rather, sleep deprivation has been shown to accelerate autoantibody production in mice,^[27]^ which could be related to the disease’s progression.

Disruptions in the biological clock, regulated by circadian clock genes such as CLOCK, BMAL-1, Reverse orientation c-ErbA gene α (Rev-Erbα), PER, and CRY genes, can lead to sleep deprivation.^[28]^ The circadian clock is known to be connected to the immune system,^[28–30]^ and any disruption could potentially contribute to the development of SADs. Circadian clock genes can influence the development of autoimmune disorders through the regulation of innate and adaptive immunity.^[29–32]^ Sleep deprivation can create a proinflammatory environment^[5]^ and has been shown to induce the expression of autoimmune-associated proteins. Increased expression of T-helper 17 (Th17) cell markers such as chemokine receptor 6, the KI67 cell proliferation marker, and cluster of differentiation 279 (CD279) apoptotic marker, as well as elevated levels of chemokine receptor 6, CXC chemokine receptor 3, and Tbet, an autoimmune-related marker in B cells, have been noted.^[33]^ The increase in inflammatory cytokines and leukocytes could heighten the potential for a breach in self-tolerance, particularly when regulatory T cells (Tregs) are dysregulated.^[34]^ CD4 + CD25 + Tregs play a critical role in preventing autoimmune disorders by maintaining self-tolerance and immune homeostasis.^[35–37]^ Reduced numbers of these cells have been observed in peripheral blood in SLE and other autoimmune diseases.^[35–37]^

The relationship between telomere shortening and autoimmune diseases has been investigated,^[38]^ and there appears to be a link between sleep deprivation and telomere shortening. One study discovered that poorer sleep quality was associated with shortened telomeres in T lymphocytes,^[39]^ while another study in Korea found that individuals with poor sleep quality experienced accelerated shortening of leukocyte telomeres over a year.^[40]^ The Baltimore Longitudinal Study of Aging found that individuals with shortened telomeres were more likely to test positive for ANA 13 years later,^[41]^ and a meta-analysis revealed significantly shorter telomere length among patients with SLE.^[42]^ However, further studies investigating the direct relationship between sleep deprivation, telomere shortening, and autoimmune disorders are needed to establish a correlation.

Psychological stress is an environmental factor implicated in the development of autoimmune diseases. A Swedish retrospective cohort study found that a clinical diagnosis of stress-related disorder increased the subsequent risk of developing autoimmune diseases.^[43]^ Another large study reported that veterans with post-traumatic stress disorder had twice the risk of developing SADs compared to veterans without psychiatric disorders.^[44]^ Psychological stress may contribute to sleep deprivation among patients with autoimmune disorders,^[21,22]^ and in turn, sleep deprivation may further exacerbate the psychosocial stress experienced by the patient. A systematic review and meta-analysis revealed a moderate correlation between depression and sleep quality in SLE patients, with a higher level of depression found in SLE patients with poor sleep quality.^[45]^ Additionally, a preliminary study found that sleep deprivation was associated with more depressive symptoms and aggravated pain among patients with SLE and fibromyalgia.^[46]^

### 3.3. Systemic autoimmune disorders implicated in circadian rhythm disruption

#### 3.3.1. Rheumatoid arthritis.

RA is an SAD characterized by inflammatory primary synovial joint involvement, causing morning joint stiffness, progressive joint damage, and loss of function.^[47]^ The majority of RA patients have anti-citrullinated protein antibodies that activate complement and recruit immune cells to the synovium, leading to cytokine, chemokine, and complement activation.^[47]^ Immune cell infiltration, along with the presence of fibroblasts, contributes to bone erosion in RA.^[47]^

Several aspects of RA are affected by disruptions in the CR. Inflammatory cytokines, such as IL-6, typically stimulate the production of corticotropin-releasing hormone in the hypothalamus, leading to corticotropin release from the pituitary glands and secretion of glucocorticoids by the adrenal gland.^[48]^ Glucocorticoids, including cortisol, and catecholamines like adrenaline and norepinephrine, exhibit circadian variations and play a significant role in RA. Dysregulation in the synthesis and secretion timing or quantity of these hormones can contribute to RA symptoms and pathogenesis. Moreover, abnormal activation of the cyclic guanosine monophosphate-adenosine monophosphate synthase-stimulator of interferon genes (cGAS-STING) signaling pathway has been implicated in the development of RA.^[49]^

Glucocorticoids, such as cortisol, are released in response to pro-inflammatory events to reduce inflammation by promoting the apoptosis of pro-inflammatory T-cells, inhibiting the production of B-cell antibodies, and reducing neutrophil migration.^[50]^ Interestingly, glucocorticoids also play a role in sleep regulation. Inflammatory activity increases during the night, and the release of inflammatory cytokines peaks in the early morning, necessitating an increase in anti-inflammatory molecules like cortisol closer to awakening. However, patients with RA often exhibit dysregulation in this cycle, with cortisol release occurring much later compared to healthy individuals.^[51,52]^ At low levels, cortisol and its glucocorticoid analogs, such as dexamethasone, are considered immunologically “permissive” due to their ability to up-regulate proinflammatory receptors.^[52]^ Abnormal hyposecretion of cortisol throughout the night in chronic diseases may explain the prevalence of early morning joint symptoms in RA patients.^[51]^ A study by Poolman et al^[53]^ also demonstrated the cyclic pattern of immune pathways, showing modulation of signal transducer and activator of transcription 3 and IL-6 levels based on the time of day.

Light stimulation induces the release of serotonin, dopamine, and cortisol, while suppressing the release of melatonin, norepinephrine, and acetylcholine.^[54]^ RA patients often exhibit significantly elevated melatonin release throughout the night, as well as increased blood levels of melatonin in the early morning, contributing to CR disruption. Inflammatory cytokines such as IFN-gamma, IL-1, and IL-6 are released at night in response to melatonin activation.^[54]^ Synovial macrophages with melatonin binding sites have been found in the synovial fluid of RA patients, and they can induce IL-12 secretion and nitric oxide production, potentially exacerbating early morning joint discomfort.^[55]^

In addition to circadian dysregulation of immune mediators, studies have revealed alterations in the expression of clock genes associated with RA. Clock genes play a role in intracellular timekeeping and contribute to the circadian control of hormones such as melatonin and cortisol.^[56]^ Lee et al^[57]^ investigated the relationship between PER2 and LPS-induced inflammation in RA, as PER2 proteins are important for modulating the mammalian CR through the transcriptional activation of CLOCK/BMAL1 via E-box motifs. The study found that PER2 expression decreased following LPS treatment in RA cells, whereas no significant changes were observed in control cells under the same conditions.^[57]^ This decrease in PER2 expression may contribute to the circadian dysregulation of inflammatory cytokines observed in RA. Emerging evidence linking the circadian cycle with immune disturbances suggests that sleep disturbances, including difficulty falling asleep, maintaining sleep, and daytime fatigue, should be considered direct manifestations of the disease rather than associated symptoms.^[58]^

#### 3.3.2. Systemic lupus erythematosus.

SLE, also known as Lupus Nephritis when it affects the kidneys, is a type of SAD. Like other autoimmune conditions, SLE involves a breakdown in self-tolerance, which can occur due to cellular damage caused by infections or other cytotoxic environmental stimuli. Exposure of the immune system to self-antigens triggers innate and adaptive responses.^[58]^ Toll-like receptors on the cell membrane can be activated by extracellular DNA and RNA, leading to the activation of IFN regulatory families, nuclear factor kappa B, and Mitogen-activated protein kinase pathways, which increase the production of proinflammatory mediators.^[58]^ This activation can stimulate neutrophils, T-lymphocytes, and B-lymphocytes.^[59]^ In SLE, T cells exhibit abnormal gene expression favoring the production of inflammatory cytokines, which promote the generation of autoreactive B cells and inhibit B-cell neutralization. The uncontrolled proliferation of stimulated B cells results in increased self-antigen presentation and production of autoantibodies, perpetuating the autoimmune response and causing tissue damage.^[59]^ Recent research has also shown that the loss of mitochondrial topoisomerase I (TOP1MT), a regulator of cytosolic mitochondrial DNA, triggers the release of mitochondrial DNA into the cytosol. This, in turn, activates the cGAS-STING innate immune signaling pathway, which is known to be active in SLE and other autoimmune diseases.^[49,60]^

SLE-associated dysregulated pathways have been linked to circadian abnormalities. Patients with SLE or those at risk of developing it often experience sleep disruptions, which significantly impact their quality of life.^[12]^ The dysregulation of T-cell activity, a hallmark of SLE, can be influenced by the CR. Natural Tregs, for instance, follow a 24-hour cycle, and sleep deprivation disrupts this rhythm, leading to impaired Treg function, which may contribute to an exacerbated autoimmune response.^[61]^ This evidence suggests that dysregulation of the CR could have a negative impact on disease progression.

The association between melatonin and SLE has been postulated, but findings from different research groups present inconsistent results. Several studies have shown lower levels of melatonin in SLE patients compared to controls, with some demonstrating a negative correlation between the severity of the condition and melatonin levels.^[62]^ Further evidence revealed a more complex regulatory role for melatonin. While melatonin has been shown to reduce exaggerated Th1 and innate inflammatory responses in healthy individuals, it has the opposite effect on cells from SLE patients.^[63]^ Melatonin has also been found to modulate the levels of IL-6 and IL-9 and the functioning of regulatory T cells through the expression of forkhead box protein 3 gene and B-cell activating factor (BAFF). Forkhead box protein 3 gene controls the growth of regulatory T cells responsible for initiating peripheral tolerance mechanisms, thus helping to prevent autoimmunity.^[64]^ BAFF, on the other hand, is produced by hematopoietic cells and is part of the TNF family of cytokines. It plays a crucial role in B-cell activation, differentiation, and survival.^[64]^

Several studies have implicated circadian clock proteins in SLE pathogenesis. Early et al focused on the associations between the BMAL1 clock protein and the immune response. BMAL1 regulates IL-1B in macrophages through the transcription factors nuclear factor erythroid 2-related factor 2.^[65]^ In patients with Lupus Nephritis, nuclear factor erythroid 2-related factor 2 expression has been found to be increased in glomeruli, potentially exacerbating the immune response. Another study demonstrated that BMAL1-knockout macrophages exhibit insufficiently sustained mitochondrial function, enhanced glycolysis, metabolic reprogramming of hypoxia-inducible factor 1-alpha-dependent processes, and inflammatory activation.^[66]^ Cao et al^[67]^ investigated the impact of genetic clock protein duplication on SLE induced by drugs or occurring naturally. Their findings showed that mice lacking CRY1 and CRY2 had higher concentrations of anti-nuclear antibodies and greater complement precipitation in glomeruli.^[67]^ Furthermore, deficiencies in CRY proteins were found to accelerate B-cell maturation in the peritoneal cavity and spleen, potentially leading to systemic effects.^[67]^ This evidence suggests that clock proteins have broader functions beyond CR regulation and illustrates their involvement in SLE.

#### 3.3.3. Inflammatory myopathies.

IM constitute a group of SADs characterized by muscle inflammation.^[68]^ IMs can be further classified into 3 distinct subtypes: Polymyositis, Dermatomyositis, and Inclusion Body Myositis (IBM).^[68]^ While the pathophysiology of each subtype varies, IM conditions generally present with similar symptoms, including muscle pain, progressive weakness, and elevated serum creatine kinase (CK) levels.^[68–70]^ Several skeletal muscle metabolic and signaling molecules have been shown to follow a rhythmic cycle.^[62,71]^ BMAL1, for instance, is expressed in skeletal muscles and regulated through BMAL1: CLOCK feedback loops.^[72]^ In macrophages, BMAL1 has been found to play an immune role by inhibiting phagocytosis, impairing immune defences, and driving the production of the proinflammatory cytokine IL-1β through the modulation of energy metabolism.^[73]^ Studies involving mice knockout experiments have described the tissue-specific activity of BMAL1, where muscle-specific restoration of BMAL1 functions corrected muscle weakness without restoring circadian behavior.^[74]^ Despite these findings, the significance of the CR in IM remains undetermined, and there is limited literature on direct circadian mechanisms.

Common causes of IM include viral infections, underlying malignancies, predisposing human leukocyte antigen variations, and certain medications such as antiepileptics and antiarrhythmics.^[75]^ In some cases, underlying systemic disorders can inappropriately activate cytotoxic T-cells against muscular antigens, leading to damage to the endomysium layer of skeletal muscle.^[68]^ Recent research has shown an increase in the cGAS-STING signaling pathway in individuals with IM, with cGAS and STING predominantly found in vascular structures, inflammatory infiltrates, and atrophic and necrotic fibers.^[76]^ These findings suggest a potential disease mechanism where fiber atrophy or necrosis is the result of IFN production from the cGAS-STING pathway.^[76]^ However, there is no consensus in the literature regarding the association between IM and disruptions in the CR. A cohort study of 13 adult patients with sporadic IBM found that sleep disturbance was more common in IBM patients compared to healthy controls.^[77]^ The pathophysiology underlying these disruptions, however, has not been proposed.

The initiation of IM is believed to involve the overactivation of the complement system, induced by pathogenic autoantibodies binding to endothelial antigens. Proper functioning of the complement system relies on the activity of circadian clock regulatory CRY proteins.^[78]^ Studies involving CRY1/CRY2 double knockout mice have shown that the absence of these proteins results in an autoimmune disease-like phenotype, with increased serum levels of complement component 3, overactivation of complement pathways, and subsequent excessive propagation of the T-cell response. Conversely, expression of the complement component 1q protein, which is normally involved in the clearance of apoptotic debris and immune complexes, has been found to be downregulated in the same animal model. Reduced complement component 1q activity has previously been implicated in the progression of SLE.^[79]^ Although there is no comprehensive experimental evidence specifically implicating circadian regulatory mechanisms in the pathogenesis of Dermatomyositis, similar to those described in SLE, it remains unclear whether these mechanisms exist in IM. Further research efforts are needed to determine whether circadian changes play a role in IM and whether interventions targeting the CR could offer therapeutic benefits.

### 3.4. Future directions in chrono-immunology and treatment strategies of SADs

#### 3.4.1. Current guidelines in SADs treatment.

The current treatment recommendations for SADs primarily involve disease-modifying agents that target immune pathways.^[47,80,81]^ The choice of treatment approach varies depending on the specific condition and the severity of the disease.^[47,51]^ Methotrexate, for example, has demonstrated efficacy in autoimmune conditions by inhibiting leukocyte proliferation through interference with nucleic acid synthesis. It is commonly prescribed for rheumatic diseases. Tofacitinib, a Janus kinase inhibitor, has been proposed as an alternative treatment for RA with similar effectiveness.^[47,51]^ TNF inhibitors, such as rituximab, are also frequently used. In cases of resistant RA, abatacept, an inhibitor of T-cell activation, may be employed.^[47]^ These anti-inflammatory drugs find application in various autoimmune conditions. Specific treatments for SLE include long-term therapy with hydroxychloroquine. Glucocorticoids are effective in rapidly managing autoimmune flares but are generally not recommended for long-term use due to their side effects and significant immune suppression.^[47,51]^

Sleep deprivation is a commonly associated symptom in SADs. Despite emerging evidence linking the CR and immune system regulatory networks, sleep deprivation is not widely accepted as a risk factor, manifestation, or prognostic measure of autoimmune conditions.^[11]^ For instance, the 2019 European League Against Rheumatism/American College of Rheumatology classification for SLE includes disease criteria such as serum antinuclear antibodies, fever, leukopenia, and psychosis.^[82]^ Although recent research has demonstrated the significance of the relationship between sleep deprivation and SLE, sleep deprivation is not listed as one of the criteria and is not prioritized in treatment. However, managing sleep disturbances in SLE is crucial as they significantly impact patients’ quality of life. Studies have shown that poor sleepers with SLE experience lower self-assessed quality of life scores, affecting various essential domains including physical and emotional health, energy levels, and intimate relationships.^[83]^ Similarly, sleep deprivation is not considered a risk or prognostic factor for RA.^[84]^ Nevertheless, studies highlight its association with increased pain levels in RA patients. A cross-sectional study by Grabovac et al found that 58% of RA patients reported suboptimal sleep, and these patients experienced higher levels of pain compared to those with optimal sleep.^[58]^

None of the currently used pharmacological approaches in autoimmune disease management have been proven effective in improving sleep quality for patients.^[85]^ However, several studies mentioned in this review emphasize the importance of better understanding the role of sleep cycle regulatory mechanisms in autoimmune conditions. Incorporating specific strategies aimed at restoring proper CR functioning could open up new avenues in the treatment of SADs.

#### 3.4.2. Circadian rhythm based approaches in the treatment of autoimmune conditions.

##### 3.4.2.1. Circadian disruption agonists.

**Figure 2.**
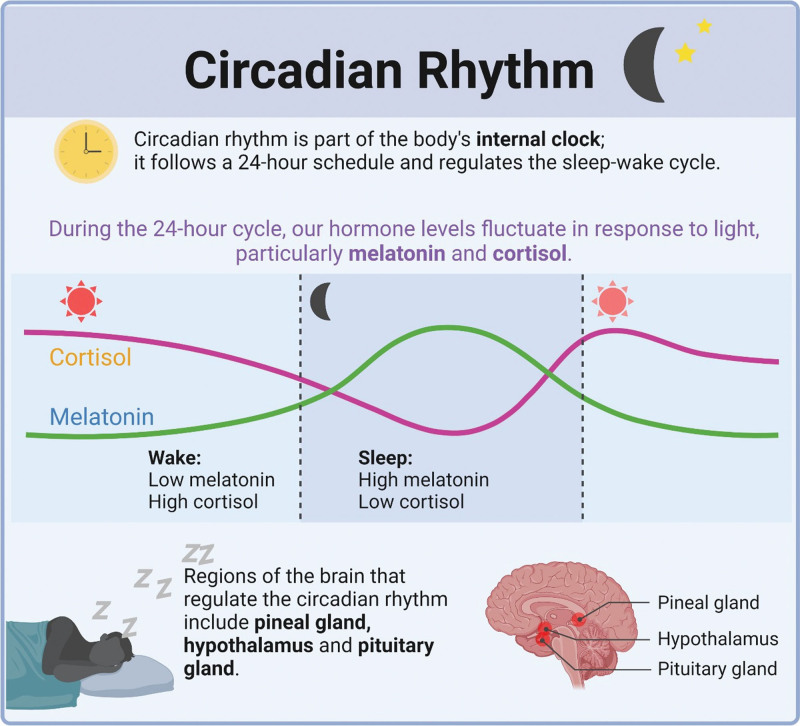
Role of melatonin and cortisol in circadian rhythm (created with BioRender.com).^[97]^

Novel drug molecules are under development to target circadian cycle disruptions in patients with SADs. One notable target is Rev-Erb, a major transcriptional factor that regulates BMAL1 expression. Several Rev-Erbα agonists, such as SR9009 and SR9011, have been extensively studied in vivo to assess their therapeutic potential (Fig. 2). Although Rev-Erbα agonists have not yet been implemented in clinical practice, studies have demonstrated their effectiveness in modulating immune responses, metabolic disorders, and cancer. For instance, a study by Wang et al showed that Rev-Erb agonists suppress the production of IL-17, a cytokine involved in inflammatory responses of psoriasiform dermatitis.^[86]^ In mouse models, topical application of SR9009 inhibited the effects of IL-17-producing gamma delta T cells, a crucial immune cell type implicated in the pathogenesis of multiple SADs such as autoimmune encephalomyelitis and RA. Subsequent studies confirmed that the depletion of Rev-Erbα increased IL-17 production from these cells, while the synthetic agonists suppressed their activity. Furthermore, pharmacological modulation of Rev-Erb activity using selective small molecules inhibited the progression of autoimmunity, demonstrating the viability of Rev-Erb targets for therapy.^[86]^ However, further research is required to establish a direct correlation between Rev-Erb factors and the regulation of IL-17-producing gamma delta T cells, highlighting the need for additional investigations to evaluate the therapeutic value of Rev-Erb agonists in other autoimmune diseases other autoimmune diseases.

Another therapeutic target for circadian disruption is a selective retinoic acid-related orphan receptor γt (RORγt) antagonist compound called TMP920, which suppresses Th17-mediated cell responses (Fig. 2).^[87,88]^ Although the benefits of RORγt antagonists have been demonstrated in in vitro and in vivo studies, it is important to note that there are currently no Food and Drug Administration-approved ROR agonists available in the market. Nonetheless, these antagonists have shown potential in suppressing immune responses within the T-cell lineage and may provide therapeutic benefits for autoimmune disorders resulting from T-cell dysfunction.^[87,88]^ Ongoing research has identified 2 novel treatments targeting ROR: 1) endogenous ROR modulators, such as oxysterol, which can bind to the RORα/γ LBD and inversely agonize the receptors, and 2) a Liver X receptor agonist (T0901317), one of the first synthetic inverse agonists for ROR (Fig. 2).^[89]^ Similarly, therapeutic strategies aimed at targeting the PER and CRY clock genes are still in the early stages, with only one study investigating the effects of small molecules on these genes. Jordan et al confirmed in vivo that peroxisome proliferator-activated receptor-δ (can downregulate the association of CRY1 and CRY2 through agonist ligand binding (Fig. 2).^[90]^ Therefore, further research is warranted to identify and evaluate more pharmacological agents for the treatment of circadian dysfunction observed in SADs.

##### 3.4.2.2. Melatonin as a potential therapeutic target.

**Figure 3.**
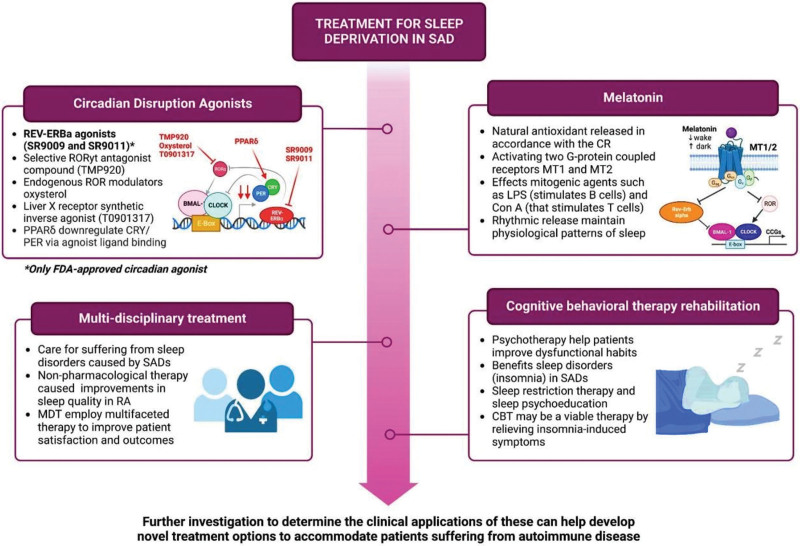
General treatment algorithm of evidence-based recommendations to treat sleep deprivation in systemic autoimmune disorders. (Original image created with biorender.com by HH). BMAL-1 = brain and muscle ARNT-like 1, CBT = cognitive behavioral therapy, CCGs = clock controlled genes, CLOCK = circadian locomotor output cycles kaput, CRY = cryptochrome circadian regulator, LPS = lipo-polysacharride, MT receptors = melatonin receptors, PER = period circadian regulator, PPARδ = peroxisome proliferator-activated receptor δ, Rev-Erb = reverse orientation c-ErbA gene α, ROR = retinoic acid related orphan receptor, SAD = systemic autoimmune disorder.

Melatonin, a natural hormone released from the pineal gland in accordance with the CR, has emerged as a potential therapeutic target. Melatonin levels vary throughout the day, with low levels during daytime and a peak at night.^[91,92]^ In addition to its role in modulating sleep, learning, and memory, melatonin acts as a neuroprotective factor in the central nervous system by activating melatonin (MT)1 and MT2 receptors, both of which are G-protein coupled receptors (Figs. 2 and 3).^[93]^ The immunomodulatory effects of melatonin and its implications for autoimmune diseases are noteworthy. Melatonin improves both innate and humoral immune responses and regulates cytokine production. It modulates immune responses through processes such as cyclic AMP signal transduction and L-type Calcium channels, which may play an anti-inflammatory role when antagonized.^[93]^ In addition to the immunostimulatory release of cytokines such as IL-1B and IFN-γ, melatonin application to antigen-primed mice has been shown to increase the production of IL-10, suggesting a possible anti-inflammatory function of melatonin.^[94]^ Other immunosuppressive actions of melatonin include inhibition of IFN-gamma, natural killer cell activity, TNF-alpha synthesis, and T-lymphocyte proliferation, indicating potential mechanisms through which melatonin exerts its immunosuppressive effects.^[95,96]^ Moreover, the rhythmic release of melatonin helps maintain physiological sleep patterns, thereby preventing sleep deprivation, which has been associated with exacerbations of autoimmune diseases such as RA and SLE.^[97]^ Understanding the complex mechanisms by which melatonin suppresses immune function can pave the way for novel therapeutic approaches to modulate aberrant immune responses. Further research on this subject can guide the application of melatonin agonists in the treatment of autoimmune diseases, representing an intriguing yet unexplored area of immunological research.

#### 3.4.3. Non-pharmacological interventions.

##### 3.4.3.1. Multidisciplinary team (MDT) approach.

In addition to novel drug therapies, non-pharmacological interventions are available to assist individuals with sleep disturbances caused by SADs (Fig. 3). Exercise interventions, for instance, have demonstrated improvements in sleep duration and quality for people with RA.^[98]^ Implementing a MDT approach is one effective intervention. Patients with chronic diseases, including SADs, often require consultations with multiple practitioners, resulting in longer wait times and delayed treatment.^[99]^ By employing an MDT, comprehensive therapy involving various specialties can be delivered in a single visit, reducing wait times and potentially improving patient health outcomes.^[99]^ This collaborative approach is particularly important when addressing sleep issues in SADs, as sleep deprivation and SADs can impact multiple organ systems and are mutually influenced. MDTs have shown improved patient outcomes in various illnesses, such as amyotrophic lateral sclerosis, heart failure, certain cancers, and sleep quality in Alzheimer’s patients.^[7,99]^ These reported benefits, including those in Alzheimer’s patients, suggest the potential usefulness of MDTs in treating sleep disorders in SAD patients. However, further research is needed to better understand their effects.

##### 3.4.3.2. Cognitive behavioral therapy (CBT).

CBT is a form of psychotherapy designed to help patients improve maladaptive behaviors (Fig. 3). It has demonstrated benefits for individuals with various conditions, including sleep disorders like insomnia.^[99]^ As previously mentioned, many individuals with SADs, such as SLE, sleep for fewer than 7 hours per night. CBT often involves a combination of treatments, such as sleep restriction, cognitive therapy, and sleep psychoeducation.^[100]^ A meta-analysis conducted by the European Sleep Research Society and the American Academy of Sleep Medicine identified CBT as the first-line therapy for insomnia and also showed promising effects in treating insomnia in patients with chronic pain, fibromyalgia, and breast cancer.^[101]^ Another meta-analysis found that insomnia-related symptoms significantly decreased in intensity 3 to 6 months after CBT treatment, although the benefits diminished over time.^[102]^ Given these findings, CBT may be a viable therapy for patients experiencing sleep difficulties caused by SADs. However, similar to the MDT approach, further study is necessary to fully understand its benefits and determine its optimal inclusion for improving patient well-being.

## 4. Conclusion

The interplay between the CR and sleep has been extensively studied in the context of immunomodulation. Sleep deprivation has been found to exacerbate common SADs such as RA and SLE, negatively impacting patients’ health and quality of life. However, there is limited data exploring the association between immune dysregulation, circadian disruption, and other SADs such as IMs, highlighting the need for further investigation in future studies. Understanding the intricate relationship between sleep physiology and autoimmune exacerbation can lead to the development of targeted pharmacological interventions. For instance, the use of CR agonists and melatonin administration could be explored as potential therapeutic options. Additionally, recognizing the value of non-pharmacological treatments such as MDT approaches and CBT in managing sleep disturbances in autoimmune diseases is essential.

Further investigation is warranted to determine the clinical applications of these interventions, ultimately leading to the development of novel treatment options that cater to the specific needs of patients suffering from autoimmune diseases. By addressing sleep disruptions and CR disturbances, significant improvements in health and outcomes can be achieved in the field of rheumatology.

## Acknowledgments

We acknowledge Icormed Research Collaborative and Toufik’s World Medical Association for their support in data collection and research facilitation.

## Author contributions

**Conceptualization:** Wireko Andrew Awuah.

**Formal analysis:** Wireko Andrew Awuah.

**Investigation:** Wireko Andrew Awuah.

**Methodology:** Wireko Andrew Awuah.

**Supervision:** Wireko Andrew Awuah, Nobendu Mukerjee, Athanasios Alexiou, Marios Papadakis.

**Visualization:** Nobendu Mukerjee, Athanasios Alexiou, Marios Papadakis.

**Writing – original draft:** Wireko Andrew Awuah, Helen Huang, Jacob Kalmanovich, Aashna Mehta, Tatiana Mikhailova, Jyi Cheng Ng, Toufik Abdul-Rahman, Favour Tope Adebusoye, Joecelyn Kirani Tan, Karl Kamanousa, Tomas Ferreira, Sakshi Roy, Mrinmoy Kundu, Rohan Yarlagadda, Nobendu Mukerjee, Athanasios Alexiou, Marios Papadakis.

**Writing – review & editing:** Wireko Andrew Awuah, Helen Huang, Jacob Kalmanovich, Aashna Mehta, Tatiana Mikhailova, Jyi Cheng Ng, Toufik Abdul-Rahman, Favour Tope Adebusoye, Joecelyn Kirani Tan, Karl Kamanousa, Tomas Ferreira, Sakshi Roy, Mrinmoy Kundu, Rohan Yarlagadda, Nobendu Mukerjee, Athanasios Alexiou, Marios Papadakis.

## References

[1] Khan S Nabi G Yao L. Health risks associated with genetic alterations in internal clock system by external factors. Int J Biol Sci. 2018;14:791–8. 29910689 10.7150/ijbs.23744 PMC6001675

[2] Reddy S Reddy V Sharma S. Physiology, Circadian Rhythm. In: StatPearls. Treasure Island, FL: StatPearls Publishing; 2022. Available at: https://www.ncbi.nlm.nih.gov/books/NBK519507/ [access date April 21, 2023]. 30137792

[3] Torres-Ruiz J Sulli A Cutolo M. Air travel, circadian rhythms/hormones, and autoimmunity. Clin Rev Allergy Immunol. 2017;53:117–25. 28244020 10.1007/s12016-017-8599-2

[4] Karagianni P Tzioufas AG. Epigenetic perspectives on systemic autoimmune disease. J Autoimmun. 2019;104:102315. 31421964 10.1016/j.jaut.2019.102315

[5] Zielinski MR Systrom DM Rose NR. Fatigue, sleep, and autoimmune and related disorders. Front Immunol. 2019;10:1827. 31447842 10.3389/fimmu.2019.01827 PMC6691096

[6] Ramos-Casals M Brito-Zerón P Kostov B. Google-driven search for big data in autoimmune geoepidemiology: analysis of 394,827 patients with systemic autoimmune diseases. Autoimmun Rev. 2015;14:670–9. 25842074 10.1016/j.autrev.2015.03.008

[7] Cooper GS Stroehla BC. The epidemiology of autoimmune diseases. Autoimmun Rev. 2003;2:119–25. 12848952 10.1016/s1568-9972(03)00006-5

[8] Tsokos GC Lo MS Reis PC. New insights into the immunopathogenesis of systemic lupus erythematosus. Nat Rev Rheumatol. 2016;12:716–30. 27872476 10.1038/nrrheum.2016.186

[9] Wang L Wang FS Gershwin ME. Human autoimmune diseases: a comprehensive update. J Intern Med. 2015;278:369–95. 26212387 10.1111/joim.12395

[10] Wahren-Herlenius M Dörner T. Immunopathogenic mechanisms of systemic autoimmune disease. Lancet. 2013;382:819–31. 23993191 10.1016/S0140-6736(13)60954-X

[11] Garbarino S Lanteri P Bragazzi NL. Role of sleep deprivation in immune-related disease risk and outcomes. Commun Biol. 2021;4:1304. 34795404 10.1038/s42003-021-02825-4 PMC8602722

[12] Young KA Munroe ME Harley JB. Less than 7 hours of sleep per night is associated with transitioning to systemic lupus erythematosus. Lupus. 2018;27:1524–31. 29804502 10.1177/0961203318778368 PMC6026567

[13] Honma S. The mammalian circadian system: a hierarchical multi-oscillator structure for generating circadian rhythm. J Physiol Sci. 2018;68:207–19. 29460036 10.1007/s12576-018-0597-5 PMC10717972

[14] Foster RG. Sleep, circadian rhythms and health. Interface Focus. 2020;10:20190098. 10.1098/rsfs.2019.0098 PMC7202392 32382406

[15] Yoshida Y Fujiki N Nakajima T. Fluctuation of extracellular hypocretin-1 (orexin A) levels in the rat in relation to the light–dark cycle and sleep–wake activities. Eur J Neurosci. 2001;14:1075–81. 11683899 10.1046/j.0953-816x.2001.01725.x

[16] Zhang S Zeitzer JM Yoshida Y. Lesions of the suprachiasmatic nucleus eliminate the daily rhythm of hypocretin-1 release. Sleep. 2004;27:619–27. 15282996 10.1093/sleep/27.4.619

[17] Yamakawa GR Brady RD Sun M. The interaction of the circadian and immune system: desynchrony as a pathological outcome to traumatic brain injury. Neurobiol Sleep Circadian Rhythms. 2020;9:100058. 33364525 10.1016/j.nbscr.2020.100058 PMC7752723

[18] Comas M Gordon CJ Oliver BG. A circadian based inflammatory response–implications for respiratory disease and treatment. Sleep Sci Pract. 2017;1:1–9.

[19] Guerrero-Vargas NN Salgado-Delgado R del Carmen Basualdo M. Reciprocal interaction between the suprachiasmatic nucleus and the immune system tunes down the inflammatory response to lipopolysaccharide. J Neuroimmunol. 2014;273:22–30. 24916044 10.1016/j.jneuroim.2014.05.012

[20] Sangle SR Tench CM D’Cruz DP. Autoimmune rheumatic disease and sleep: a review. Curr Opin Pulm Med. 2015;21:553–6. 26402614 10.1097/MCP.0000000000000215

[21] Palagini L Tani C Mauri M. Sleep disorders and systemic lupus erythematosus. Lupus. 2014;23:115–23. 24421291 10.1177/0961203313518623

[22] Zhao Q Deng N Chen S. Systemic lupus erythematosus is associated with negatively variable impacts on domains of sleep disturbances: a systematic review and meta-analysis. Psychol Health Med. 2018;23:685–97. 29488396 10.1080/13548506.2018.1442011

[23] Kok VC Horng JT Hung GD. Risk of autoimmune disease in adults with chronic insomnia requiring sleep-inducing pills: a population-based longitudinal study. J Gen Intern Med. 2016;31:1019–26. 27130621 10.1007/s11606-016-3717-z PMC4978676

[24] Chung WS Lin CL Kao CH. Association of systemic lupus erythematosus and sleep disorders: a nationwide population-based cohort study. Lupus. 2016;25:382–8. 26585071 10.1177/0961203315617843

[25] Hsiao YH Chen YT Tseng CM. Sleep disorders and increased risk of autoimmune diseases in individuals without sleep apnea. Sleep. 2015;38:581–6. 25669189 10.5665/sleep.4574 PMC4355897

[26] Sang N Gao RC Zhang MY. Causal relationship between sleep traits and risk of systemic lupus erythematosus: a two-sample Mendelian randomization study. Front Immunol. 2022;13:918749. 35784289 10.3389/fimmu.2022.918749 PMC9248809

[27] Palma BD Gabriel A Jr Colugnati FA. Effects of sleep deprivation on the development of autoimmune disease in an experimental model of systemic lupus erythematosus. Am J Physiol Regul Integr Comp Physiol. 2006;291:R1527–32. 16809486 10.1152/ajpregu.00186.2006

[28] Rijo-Ferreira F Takahashi JS. Genomics of circadian rhythms in health and disease. Genome Med. 2019;11:1–6. 31847894 10.1186/s13073-019-0704-0 PMC6916512

[29] Sutton CE Finlay CM Raverdeau M. Loss of the molecular clock in myeloid cells exacerbates T cellmediated CNS autoimmune disease. Nat Commun. 2017;8:1923. 29234010 10.1038/s41467-017-02111-0 PMC5727202

[30] Scheiermann C Gibbs J Ince L. Clocking in to immunity. Nat Rev Immunol. 2018;18:423–37. 29662121 10.1038/s41577-018-0008-4

[31] Gibbs JE Ray DW. The role of the circadian clock in rheumatoid arthritis. Arthritis Res Ther. 2013;15:205–9. 23427807 10.1186/ar4146 PMC3672712

[32] Yoshida K Hashimoto T Sakai Y. Involvement of the circadian rhythm and inflammatory cytokines in the pathogenesis of rheumatoid arthritis. J Immunol Res. 2014;2014:282495. 24901009 10.1155/2014/282495 PMC4034483

[33] Liu X Chen B Huang Z. Effects of poor sleep on the immune cell landscape as assessed by single-cell analysis. Commun Biol. 2021;4:1325. 34824394 10.1038/s42003-021-02859-8 PMC8617259

[34] Gravano DM Hoyer KK. Promotion and prevention of autoimmune disease by CD8+ T cells. J Autoimmun. 2013;45:68–79. 23871638 10.1016/j.jaut.2013.06.004

[35] Grant CR Liberal R Mieli-Vergani G. Regulatory T-cells in autoimmune diseases: challenges, controversies and – yet – unanswered questions. Autoimmun Rev. 2015;14:105–16. 25449680 10.1016/j.autrev.2014.10.012

[36] Shu Y Hu Q Long H. Epigenetic variability of CD4+ CD25+Tregs contributes to the pathogenesis of autoimmune diseases. Clin Rev Allergy Immunol. 2017;52:260–72. 27687891 10.1007/s12016-016-8590-3

[37] Kumar P Saini S Khan S. Restoring self-tolerance in autoimmune diseases by enhancing regulatory T-cells. Cell Immunol. 2019;339:41–9. 30482489 10.1016/j.cellimm.2018.09.008 PMC6440877

[38] Goronzy JJ Weyand CM. Immune aging and autoimmunity. Cell Mol Life Sci. 2012;69:1615–23. 22466672 10.1007/s00018-012-0970-0 PMC4277694

[39] Prather AA Gurfein B Moran P. Tired telomeres: poor global sleep quality, perceived stress, and telomere length in immune cell subsets in obese men and women. Brain Behav Immun. 2015;47:155–62. 25535858 10.1016/j.bbi.2014.12.011 PMC4468027

[40] Jin JH Kwon HS Choi SH. Association between sleep parameters and longitudinal shortening of telomere length. Aging (Albany NY). 2022;14:2930–44. 35366243 10.18632/aging.203993 PMC9037260

[41] Meier HC Parks CG Liu HB. Cellular aging over 13 years associated with incident antinuclear antibody positivity in the Baltimore Longitudinal Study of Aging. J Autoimmun. 2019;105:102295. 31303354 10.1016/j.jaut.2019.06.006 PMC6878149

[42] Lee YH Jung JH Seo YH. Association between shortened telomere length and systemic lupus erythematosus: a meta-analysis. Lupus. 2017;26:282–8. 27510600 10.1177/0961203316662721

[43] Frazzei G van Vollenhoven RF de Jong BA. Preclinical autoimmune disease: a comparison of rheumatoid arthritis, systemic lupus erythematosus, multiple sclerosis and type 1 diabetes. Front Immunol. 2022;13:899372. 35844538 10.3389/fimmu.2022.899372 PMC9281565

[44] O’Donovan A Cohen BE Seal KH. Elevated risk for autoimmune disorders in Iraq and Afghanistan veterans with posttraumatic stress disorder. Biol Psychiatry. 2015;77:365–74. 25104173 10.1016/j.biopsych.2014.06.015 PMC4277929

[45] Yin R Li L Xu L. Association between depression and sleep quality in patients with systemic lupus erythematosus: a systematic review and metaanalysis. Sleep Breath. 2022;26:429–41. 34032968 10.1007/s11325-021-02405-0 PMC8857107

[46] Cervilla O Miró E Martínez MP. Sleep quality and clinical and psychological manifestations in women with mild systemic lupus erythematosus activity compared to women with fibromyalgia: a preliminary study. Mod Rheumatol. 2020;30:1016–24. 31599659 10.1080/14397595.2019.1679973

[47] Chauhan K Jandu JS Goyal A Al-Dhahir MA. Rheumatoid srthritis. In: StatPearls. Treasure Island, FL: StatPearls Publishing; 2022. Available at: https://www.ncbi.nlm.nih.gov/books/NBK441999/ [access date May 05, 2023].

[48] Cutolo M Villaggio B Otsa K. Altered circadian rhythms in rheumatoid arthritis patients play a role in the disease’s symptoms. Autoimmun Rev. 2005;4:497–502. 16214085 10.1016/j.autrev.2005.04.019

[49] Hu Y Chen B Yang F. Emerging role of the cGAS-STING signaling pathway in autoimmune diseases: Biologic function, mechanisms and clinical prospection. Autoimmun Rev. 2022;21:103155. 35902046 10.1016/j.autrev.2022.103155

[50] Thau L Gandhi J Sharma S. Physiology, cortisol. In: StatPearls. Treasure Island, FL: StatPearls Publishing; 2021. Available at: https://www.ncbi.nlm.nih.gov/books/NBK538239/ [access date May 05, 2023]. 30855827

[51] Cutolo M. Circadian rhythms and rheumatoid arthritis. Joint Bone Spine. 2019;86:327–33. 30227223 10.1016/j.jbspin.2018.09.003

[52] Rao RT Pierre KK Schlesinger N. The potential of circadian realignment in rheumatoid arthritis. Crit Rev Biomed Eng. 2016;44:177–91. 28605351 10.1615/CritRevBiomedEng.2016018812 PMC5915622

[53] Poolman TM Gibbs J Walker AL. Rheumatoid arthritis reprograms circadian output pathways. Arthritis Res Ther. 2019;21:1–3. 30728072 10.1186/s13075-019-1825-y PMC6366099

[54] Scherholz ML Schlesinger N Androulakis IP. Chronopharmacology of glucocorticoids. Adv Drug Deliv Rev. 2019;151–152:245–61. 10.1016/j.addr.2019.02.004 PMC6703983 30797955

[55] Xia Y Chen S Zeng S. Melatonin in macrophage biology: current understanding and future perspectives. J Pineal Res. 2019;66:e12547. 30597604 10.1111/jpi.12547

[56] Charrier A Olliac B Roubertoux P. Clock genes and altered sleep–wake rhythms: their role in the development of psychiatric disorders. Int J Mol Sci. 2017;18:938. 28468274 10.3390/ijms18050938 PMC5454851

[57] Lee H Nah SS Chang SH. PER2 is downregulated by the LPS-induced inflammatory response in synoviocytes in rheumatoid arthritis and is implicated in disease susceptibility. Mol Med Rep. 2017;16:422–8. 28498398 10.3892/mmr.2017.6578

[58] Grabovac I Haider S Berner C. Sleep quality in patients with rheumatoid arthritis and associations with pain, disability, disease duration, and activity. J Clin Med. 2018;7:336. 30304765 10.3390/jcm7100336 PMC6210607

[59] Justiz Vaillant AA Goyal A Varacallo M. Systemic lupus erythematosus. In: StatPearls. Treasure Island, FL: StatPearls Publishing; 2022. Available at: https://www.ncbi.nlm.nih.gov/books/NBK535405/ [access date May 05, 2023]. 30571026

[60] Khatib IA Deng J Lei Y. Activation of the cGAS-STING innate immune response in cells with deficient mitochondrial topoisomerase TOP1MT. Hum Mol Genet. 2023;32:2422–40. 37129502 10.1093/hmg/ddad062 PMC10360396

[61] Mohandas R Douma LG Scindia Y. Circadian rhythms and renal pathophysiology. J Clin Investig. 2022;132:e148277. 35104800 10.1172/JCI148277 PMC8803319

[62] Rasheed AB Daoud MS Gorial FI. Diagnostic utility of serum melatonin levels in systemic lupus erythematosus: a case-control study. Reumatismo. 2017;69:170–4. 29320843 10.4081/reumatismo.2017.998

[63] Medrano-Campillo P Sarmiento-Soto H Álvarez-Sánchez N. Evaluation of the immunomodulatory effect of melatonin on the T-cell response in peripheral blood from systemic lupus erythematosus patients. J Pineal Res. 2015;58:219–26. 25612066 10.1111/jpi.12208

[64] Möckel T Basta F Weinmann-Menke J. B cell activating factor (BAFF): structure, functions, autoimmunity and clinical implications in systemic lupus erythematosus (SLE). Autoimmun Rev. 2021;20:102736. 33333233 10.1016/j.autrev.2020.102736

[65] Early JO Menon D Wyse CA. Circadian clock protein BMAL1 regulates IL-1β in macrophages via NRF2. Proc Natl Acad Sci USA. 2018;115:E8460–8. 30127006 10.1073/pnas.1800431115 PMC6130388

[66] Alexander RK Liou YH Knudsen NH. Bmal1 integrates mitochondrial metabolism and macrophage activation. Elife. 2020;9:e54090. 32396064 10.7554/eLife.54090 PMC7259948

[67] Cao Q Zhao X Bai J. Circadian clock cryptochrome proteins regulate autoimmunity. Proc Natl Acad Sci USA. 2017;114:12548–53. 29109286 10.1073/pnas.1619119114 PMC5703267

[68] Mukerjee N. A brief review on the overview on immunology of COVID-19: current state of the research. Int J Sci Res. 2020;9:SR201102135538.

[69] Zong M Lundberg IE. Pathogenesis, classification and treatment of inflammatory myopathies. Nat Rev Rheumatol. 2011;7:297–306. 21468145 10.1038/nrrheum.2011.39

[70] Zhang R Lahens NF Ballance HI. A circadian gene expression atlas in mammals: implications for biology and medicine. Proc Natl Acad Sci USA. 2014;111:16219–24. 25349387 10.1073/pnas.1408886111 PMC4234565

[71] Hodge BA Wen Y Riley LA. The endogenous molecular clock orchestrates the temporal separation of substrate metabolism in skeletal muscle. SkeletMuscle. 2015;5:1–6. 10.1186/s13395-015-0039-5 PMC4440511 26000164

[72] Riley LA Esser KA. The role of the molecular clock in skeletal muscle and what it is teaching us about muscle-bone crosstalk. Curr Osteoporos Rep. 2017;15:222–30. 28421465 10.1007/s11914-017-0363-2 PMC5442191

[73] Kitchen GB Cunningham PS Poolman TM. The clock gene Bmal1 inhibits macrophage motility, phagocytosis, and impairs defense against pneumonia. Proc Natl Acad Sci USA. 2020;117:1543–51. 31900362 10.1073/pnas.1915932117 PMC6983378

[74] McDearmon EL Patel KN Ko CH. Dissecting the functions of the mammalian clock protein BMAL1 by tissue-specific rescue in mice. Science. 2006;314:1304–8. 17124323 10.1126/science.1132430 PMC3756687

[75] Sarwar A Dydyk AM Jatwani S. Polymyositis. In: StatPearls. Treasure Island, FL: StatPearls Publishing; 2022. Available at: https://www.ncbi.nlm.nih.gov/books/NBK563129/ [access date May 05, 2023].

[76] Zhou M Cheng X Zhu W. Activation of cGAS-STING pathway – a possible cause of myofiber atrophy/necrosis in dermatomyositis and immune-mediated necrotizing myopathy. J Clin Lab Anal. 2022;36:e24631. 36030554 10.1002/jcla.24631 PMC9550984

[77] Della Marca G Sancricca C Losurdo A. Sleep disordered breathing in a cohort of patients with sporadic inclusion body myositis. Clin Neurophysiol. 2013;124:1615–21. 23583020 10.1016/j.clinph.2013.03.002

[78] Crowson AN Magro CM. The role of microvascular injury in the pathogenesis of cutaneous lesions of dermatomyositis. Hum Pathol. 1996;27:15–9. 8543305 10.1016/s0046-8177(96)90132-x

[79] Gibbs JE Blaikley J Beesley S. The nuclear receptor REV-ERBα mediates circadian regulation of innate immunity through selective regulation of inflammatory cytokines. Proc Natl Acad Sci USA. 2012;109:582–7. 22184247 10.1073/pnas.1106750109 PMC3258648

[80] Fanouriakis A Kostopoulou M Alunno A. 2019 update of the EULAR recommendations for the management of systemic lupus erythematosus. Ann Rheum Dis. 2019;78:736–45. 30926722 10.1136/annrheumdis-2019-215089

[81] Zhao M Wu J Wu H. Clinical treatment options in scleroderma: recommendations and comprehensive review. Clin Rev Allergy Immunol. 2021;62:273–91. 33449302 10.1007/s12016-020-08831-4

[82] Aringer M Costenbader K Daikh D. 2019 European League Against Rheumatism/American College of Rheumatology classification criteria for systemic lupus erythematosus. Arthritis Rheumatol. 2019;71:1400–12. 31385462 10.1002/art.40930 PMC6827566

[83] Mirbagher L Gholamrezaei A Hosseini N. Sleep quality in women with systemic lupus erythematosus: contributing factors and effects on health-related quality of life. Int J Rheum Dis. 2016;19:305–11. 24910903 10.1111/1756-185X.12418

[84] Mankia K Siddle HJ Kerschbaumer A. EULAR points to consider for conducting clinical trials and observational studies in individuals at risk of rheumatoid arthritis. Ann Rheum Dis. 2021;80:1286–98. 34362746 10.1136/annrheumdis-2021-220884 PMC8458095

[85] Reynolds AC Marshall NS Hill CL. Systematic review of the efficacy of commonly prescribed pharmacological treatments for primary treatment of sleep disturbance in patients with diagnosed autoimmune disease. Sleep Med Rev. 2020;49:101232. 31911367 10.1016/j.smrv.2019.101232

[86] Ruan W Yuan X Eltzschig HK. Circadian rhythm as a therapeutic target. Nat Rev Drug Discovery. 2021;20:287–307. 33589815 10.1038/s41573-020-00109-w PMC8525418

[87] Xiao S Yosef N Yang J. Small-molecule RORγt antagonists inhibit T helper 17 cell transcriptional network by divergent mechanisms. Immunity. 2014;40:477–89. 24745332 10.1016/j.immuni.2014.04.004 PMC4066874

[88] Park TY Park SD Cho JY. RORγt-specific transcriptional interactomic inhibition suppresses autoimmunity associated with TH17 cells. Proc Natl Acad Sci USA. 2014;111:18673–8. 25527718 10.1073/pnas.1413687112 PMC4284575

[89] Kumar N Solt LA Conkright JJ. The benzenesulfoamide T0901317 [N-(2, 2, 2-trifluoroethyl)-N-[4-[2,2, 2-trifluoro-1-hydroxy-1-(trifluoromethyl) ethyl] phenyl]-benzenesulfonamide] is a novel retinoic acid receptor-related orphan receptor-α/γ inverse agonist. Mol Pharmacol. 2010;77:228–36. 19887649 10.1124/mol.109.060905 PMC2812071

[90] Jordan SD Kriebs A Vaughan M. CRY1/2 selectively repress PPARδ and limit exercise capacity. Cell Metab. 2017;26:243–255.e6. 28683290 10.1016/j.cmet.2017.06.002 PMC5546250

[91] Liu J Clough SJ Hutchinson AJ. MT1 and MT2 melatonin receptors: a therapeutic perspective. Annu Rev Pharmacol Toxicol. 2016;56:361–83. 26514204 10.1146/annurev-pharmtox-010814-124742 PMC5091650

[92] Begum R Mamun-Or-Rashid AN Lucy TT. Potential therapeutic approach of melatonin against omicron and some other variants of SARS-CoV-2. Molecules. 2022;27:6934. 36296527 10.3390/molecules27206934 PMC9609612

[93] Srinivasan V Maestroni GJ Cardinali DP. Melatonin, immune function and aging. Immunity Ageing. 2005;2:1–0. 16316470 10.1186/1742-4933-2-17 PMC1325257

[94] Raghavendra V Singh V Shaji AV. Melatonin provides signal 3 to unprimed CD4+ T cells but failed to stimulate LPS primed B cells. Clin Exp Immunol. 2001;124:414–22. 11472402 10.1046/j.1365-2249.2001.01519.x PMC1906083

[95] Castrillon PO Esquifino AI Varas A. Effect of melatonin treatment on 24-h variations in responses to mitogens and lymphocyte subset populations in rat submaxillary lymph nodes. J Neuroendocrinol. 2000;12:758–65. 10929088 10.1046/j.1365-2826.2000.00519.x

[96] Maestroni GJ. The immunotherapeutic potential of melatonin. Expert Opin Investig Drugs. 2001;10:467–76. 10.1517/13543784.10.3.467 11227046

[97] Hickie IB Naismith SL Robillard R. Manipulating the sleepwake cycle and circadian rhythms to improve clinical management of major depression. BMC Med. 2013;11:1–27. 23521808 10.1186/1741-7015-11-79 PMC3760618

[98] McKenna SG Donnelly A Esbensen BA. The feasibility of an exercise intervention to improve sleep (time, quality and disturbance) in people with rheumatoid arthritis: a pilot RCT. Rheumatol Int. 2021;41:297–310. 33386901 10.1007/s00296-020-04760-9

[99] Shelgikar AV Durmer JS Joynt KE. Multidisciplinary sleep centers: strategies to improve care of sleep disorders patients. J Clin Sleep Med. 2014;10:693–7. 24932153 10.5664/jcsm.3808 PMC4031414

[100] Anderson KN. Insomnia and cognitive behavioural therapy – how to assess your patient and why it should be a standard part of care. J Thorac Dis. 2018;10(Suppl 1):S94–S102. 29445533 10.21037/jtd.2018.01.35 PMC5803038

[101] Chand SP Kuckel DP Huecker MR. Cognitive Behavior Therapy. In: StatPearls. Treasure Island, FL: StatPearls Publishing; 2022. Available at: https://www.ncbi.nlm.nih.gov/books/NBK470241/. [access date May 05, 2023]. 29261869

[102] van der Zweerde T Bisdounis L Kyle SD. Cognitive behavioral therapy for insomnia: a meta-analysis of long-term effects in controlled studies. Sleep Med Rev. 2019;48:101208. 31491656 10.1016/j.smrv.2019.08.002

